# Situated Psychopathology

**DOI:** 10.1007/s12124-025-09913-8

**Published:** 2025-04-28

**Authors:** Svend Brinkmann

**Affiliations:** Department of Communication and Psychology, Aalborg University, Aalborg, Denmark

**Keywords:** Diagnoses, Psychiatry, Psychopathology, Symptoms, Context, Situated

## Abstract

This article seeks to formulate a situated approach to mental disorder that overcomes some of the problems of contemporary diagnostic psychiatry. A framework is needed that aims to integrate neuroscientific knowledge about the brain and other aspects of the person with knowledge about the environment. Inspired by the work of researchers such as Thomas Fuchs, Jerome Wakefield, and Dorte Gannik, I articulate four basic principles for a theory of psychopathology as situated, which hopefully point in this direction. These principles state that a theory of psychopathology as situated is relational; that it needs a concept of ecosocial niches; that it has an externalist component; and that it sees the brain as a social organ. The article begins by providing a brief overview of some of the criticism that has recently been leveled at the expanding diagnostic psychiatry from neuroscientific and contextual approaches, and the whole point of integrating these in a situated approach to mental disorder is to find theoretical room for factors related to the brain, mind, and body of the person as well as for the adversities that people are exposed to in their lives.

## Introduction

The recent history of psychiatry has been characterized by a significant diagnostic expansion.^1^ Since the etiological model of mental disorder was replaced by the symptom model around 1980, new diagnoses have continuously been added to the current manuals, most recently with DSM-5 (in the US) and ICD-11 (which is published by the World Health Organization). In this article, I will first unfold some of the criticism that has been leveled at the expanding diagnostic psychiatry from two opposing perspectives: One represented by a neuroscientific approach known as RDoC (Research Domain Criteria), which argues that psychiatry must move beyond symptoms and find the causes of mental illness in the brain (Insel et al., 2010), and another represented by a contextual approach known as PTMF (Power Threat Meaning Framework), which argues that mental illness must be understood in light of what people are exposed to in their lives (Boyle & Johnstone, 2020) (see also Brinkmann, 2024, where I attempted a first juxtaposition of these perspectives). The contextual criticism in particular highlights the problems with a non-situated approach to psychiatry. However, my purpose is not only critical, because I want to argue more constructively that we need a situated perspective on mental illness and disorder that integrates neuroscientific knowledge about the brain and other aspects of the person with knowledge about the environment. Based on researchers such as Thomas Fuchs, Jerome Wakefield, and Dorte Gannik, I therefore develop four basic principles for a theory of psychopathology as situated. These principles are:

1. A theory of psychopathology as situated is relational.
2. A theory of psychopathology as situated needs a concept of ecosocial niches.
3. A theory of psychopathology as situated has an externalist component.
4. A theory of psychopathology as situated sees the brain as a social organ.

The starting point for the article is thus that diagnostic psychiatry has ended up in a dead end with its focus on symptoms, and that a fruitful way forward for researchers, practitioners, and most importantly the patients who need help, will go via a situated understanding of psychopathology.

### From Etiology to Symptoms

It is well known that a revolution took place in psychiatry around 1980 with the creation of DSM-III. A new psychiatric manual based on symptoms replaced the older etiological understanding of mental illness (Horwitz, 2002; Kutchins & Kirk, 1997). Before DSM-III, a diagnosis was formulated based on the patient’s biography, experiences, personality, actions, and relationships, and psychiatrists often used theoretical knowledge from psychoanalysis to describe the patient. However, this diagnostic practice was quite unreliable, prompting the switch to the subsequent diagnostic approach. With the introduction of the new approach, a diagnosis could be formulated if the patient had at least x number of symptoms from a given list within y weeks or months (depending on the specific diagnosis category). A psychiatrist would normally also seek to map significant factors in the patient’s history and experiences (the so-called anamnesis), but so much emphasis was placed on symptomatology that Horwitz refers to this entire transition as one in which etiological psychiatry was simply replaced by “diagnostic psychiatry” (Horwitz, 2002).

With the transition to the latest manual in American psychiatry, the DSM-5 from 2013, many expected a new revolution in psychiatry that would abandon the often-criticized categorical approach, where one either has or does not have a mental disorder based on the number and severity of symptoms, in favor of a dimensional approach where all people can in principle be placed somewhere on the continua. But efforts to construct a dimensional system failed, and instead the chapters of the manual were simply reorganized. The similarity between the two editions of the DSM means that many of the criticisms leveled at DSM-III and DSM-IV (e.g., by Kutchins & Kirk, 1997) still apply to DSM-5, and ironically now is expressed by people such as Allen Frances, who chaired the task force behind DSM-IV (Frances, 2013). So overall, it is still a symptom-based approach that dominates, which also applies to the ICD system of WHO, which has now reached ICD-11 (see Brinkmann, 2024).

In many countries around the world, there is heated debate about the increase in the number of people being diagnosed by psychiatrists. Are there more mentally ill people than before? Have we finally succeeded in developing diagnostic tools to find those afflicted that may have always existed unnoticed? Or are we stuck with a problem of over-diagnosing and pathologizing forms of human behavior and experiences that were previously considered normal (Jønsson & Brodersen, 2022)? Perhaps all three analyses are valid, depending on which area of ​​psychiatry we are talking about, because we should bear in mind that psychiatry is not a single, monolithic entity, but covers a large number of very different conditions and problems for human beings. However, it is likely that the transition to a symptom-based psychiatry in itself has been a significant driving force in the diagnostic expansion (Horwitz, 2002). This is a process with different aspects: Not only do more and more people receive a psychiatric diagnosis among the existing ones, but new diagnoses are also continuously proposed, some of which end up entering the official manuals, while others remain on the fringes of clinical practice. In ICD-11, Prolonged Grief Disorder was added to the manual, which has caused quite a stir, as grief was probably one of the last painful human conditions that existed beyond the diagnostic gaze (Brinkmann, 2018a). Furthermore, and adding to the diagnostic expansion, there is also a treatment gap that for most mental disorders is believed to be more than 50% (and for some, such as substance abuse, considerably higher), which means that more than half of those who suffer from a mental disorder are still not treated (Kohn et al., 2004). References to the treatment gap are often used by patient organizations, researchers, professionals, and the pharmaceutical industry to support the view that more should be done to find and treat the mentally ill among us. The diagnoses play a key role in this debate because they define what mental illness is and how it must be tracked down.

### Viewing Symptoms as Diseases

More and more researchers seem to agree that psychiatry’s basic idea of ​​mental illness is problematic. Alastair Morgan has formulated the idea in this way: “that there are discrete mental illnesses with clear boundaries that we should endeavor to codify and classify” (Morgan, 2015, p. 157). Morgan refers to several critical positions which have criticized the idea in different ways, not least based on its lack of understanding of how mental illness is *situated* in a person’s life and environment. Diagnostic psychiatry becomes “de-situated” when it abstracts symptoms from their contexts and sees them as illnesses and disorders in themselves. Nesse (2020), a recognized medical researcher and clinician who, among other things, is a key figure behind the development of an evolutionary approach to medicine, claims that, conversely, in somatic medicine, symptoms are typically viewed as ways in which an organism protects itself from disease. It may be through coughing or vomiting to remove a harmful substance, fever to attack pathogens, pain as a functional warning sign of danger of tissue damage, etc. But diagnostic psychiatry has done the exact opposite and identified symptoms with mental illness or disorder. In short: In psychiatry, symptoms are typically not seen as ways in which an organism tries to protect itself or get better, but rather as ways in which it gets sicker or even as mental illness in itself. Nesse refers to this as the fallacy of Viewing Symptoms as Diseases (VSAD).

Nesse perceives VSAD as a kind of fundamental attribution error, where the meaning of situations is ignored, and a psychological problem is instead attributed to the characteristics of the isolated individual, in this case in the form of symptoms, so “instead of searching for what might be arousing anxiety or low mood, many clinicians instead assume that they are pathological products of a broken brain or distorted thinking.” (p. 47). When symptoms are seen as diseases in themselves, they (the symptoms) are taken out of the contexts in which they could have made sense. This has long been criticized by Wakefield (1992), who claims that mental disorders are something more than the presence of symptoms. In his eyes, distress is only an expression of a bona fide mental disorder if there is some form of dysfunction in the mental system involved in one way or another, which I will return to below. Recognizing VSAD as the key problem for a non-situated diagnostic psychiatry does not help us very much in itself. We need to look for alternatives, and there are roughly two main contenders that try to move beyond symptoms, but in diametrically opposite directions: The neuroscientific solution that claims that we need to move beyond symptoms to find the cause of poor mental health in the brain, and the contextual approach that argues that mental illness is about people reacting to life’s events and circumstances (Brinkmann, 2024). Let us briefly look at two examples.

### Moving Beyond Symptoms: Two Directions

The first direction can be associated with the name Thomas Insel, who was the head of the National Institute of Mental Health in the US from 2002 to 2015. Given this role, it was notable when he expressed great displeasure with the diagnostic expansion of the DSM-5. As an alternative way forward, he and colleagues proposed a new classification framework for mental disorders, based on brain research and genetics, intended to inform diagnosis and treatment. This framework was called the *Research Domain Criteria* (RDoC), and an influential programmatic text was published in 2010, expressing concern with the validity of the diagnostic expansion (Insel et al., 2010):

Diagnostic categories based on clinical consensus fail to align with findings emerging from clinical neuroscience and genetics. The boundaries of these categories have not been predictive of treatment response. And, perhaps most importantly, these categories, based upon presenting signs and symptoms, may not capture fundamental underlying mechanisms of dysfunction. (Insel et al., 2010, p. 748).

According to Insel and colleagues, the consequence of a focus on symptoms at the expense of “underlying mechanisms of dysfunction” has been a very slow development of new treatments aimed at basic pathophysiological mechanisms. In short, researchers and practitioners have stared blindly at symptoms and forgotten to explore what the symptoms are symptoms of. The RDoC approach is therefore intended as a fresh start for psychiatry based on a central biological assumption, namely that mental illnesses are brain disorders (Insel et al., 2010, p. 749). The RDoC approach assumes that dysfunctions in neural circuits can be identified clinically and is based on the premise that data from genetics and clinical neuroscience will provide what the researchers call “biosignatures” as a basis for clinical treatment. However, what has been gained in terms of clinical returns is still limited, as no valid biomarkers have so far been found in psychiatry (Singh & Rose, 2009; Rose & Abi-Rached, 2013). A recent review of psychiatry’s search for biomarkers concluded: “As set out in this review, there are several proteins, metabolites and genes that have been linked with certain neuropsychiatric diseases mainly due to the advance in ‘omics’ technologies. However, none of them have demonstrated to be a real and useful biomarker in clinical practice.” (García-Gutiérrez et al., 2020). However, this does not mean that genes, hormones, the brain and the central nervous system are unimportant for mental illness and disorder. It just means that the brain, for example, is only important in the context of a living body, connected to various life projects of the person in ecological niches. The brain is precisely important if seen in a situated perspective, which I will return to below. Fuchs (2021) has rightly noted that it is not the brain that performs the various life functions of living people, but rather that it is these functions that primarily affect the functioning of the brain. The brain is an essential organ that mediates life processes but is not itself an actor or site of mental processes. The neuroscientific approach is certainly important as a critical voice within the biomedical sciences, as it counters both the diagnostic expansion and the tendency to see symptoms as diseases, but it is insufficient in itself to provide a full understanding of mental disorders, as it typically reduces these to brain processes.

From a completely different angle, *The Power Threat Meaning Framework* (PTMF) offers an equally sweeping critique of diagnostic psychiatry and paves the way for a different direction in the future. PTMF offers a contextual understanding of mental health problems, which is well expressed by Rose (2019) (although he is not a part of the PTMF movement). He believes that psychiatry now needs:

… an account that seeks to make sense of a person’s current difficulties in terms of aspects of their current situation, for example, their relationships, their experiences at work or in unemployment, their housing and financial situation, and indeed their own ways of making sense of their situation, and accounting for their distress. (Rose, 2019, p. 187).

As an alternative to symptom-based diagnostics, Rose calls for “formulation”, which is the construction of a complex story about the origin of the disorder currently experienced by a human being (Rose, 2019, p. 91). Within PTMF, this concern is expressed by insisting that the standard psychiatric approach (based on the question: “What is wrong with you?”, which is commonly answered by counting symptoms) should be replaced by the question: “What has happened to you?” (Boyle & Johnstone, 2020, p. 3). The latter question recognizes that mental health problems are typically the result of complex events in the lives, environments, and relationships that people have experienced, which should prompt a shift in clinical attention from symptoms and diagnoses to living conditions and life histories. The framework is called PTMF because it deals with what has happened (how *power* has worked in a person’s life), how the situation affected the person (*threats*), and how the person can make sense of what happened in order to survive (*meaning*).

Understanding people’s problems in light of their stories is thought to reduce stigma and increase solidarity with those affected. PTMF also explains the comorbidity that is often observed in psychiatry, namely that many people who are given a depression diagnosis will also, for example, meet the criteria for an anxiety disorder. The reason, according to PTMF, is not that such individuals suffer from two distinct mental disorders, but quite simply that the kinds of circumstances that make us feel very anxious and worried can also make us feel hopeless and despairing (Boyle & Johnstone, 2020, p. 24). It is an epidemiological fact that all forms of psychological hardship increase the risk of all types of mental disorder (p. 105), which means that the most humane and effective way to reduce suffering and distress will be to improve people’s living conditions. Reducing economic and social inequality is probably the most effective step we can take to improve population well-being, especially for groups with little power (p. 169).

PTMF is a welcome alternative to diagnostic understandings of human suffering. Rather than abstracting experiences out of their context and turning them into the “symptoms” of an unsituated individual, PTMF insists on understanding suffering and distress in the light of what people have been through. Instead of formulating more and more diagnoses, PTMF focuses on eliminating the threats in people’s life contexts. With this we are approaching a situated perspective on psychopathology, but nevertheless it seems to me that the ways in which threats, abuse, and power can harm a person’s mind and brain are somewhat downplayed by PTMF. The framework does not pay much attention to the ways in which injury to an individual can actually be carried across life contexts, because something may be damaged in the person’s brain or psyche. Just as the neuroscientific approach ran the risk of reducing mental illness to what goes on in the brain, PTMF should be careful not to dissolve the personal components of disorders into a sea of ​​contextual factors. The PTMF itself states that problems resulting from neurodegenerative disorders (e.g. dementia) or neurodivergent conditions such as autism have not been considered by the framework (Boyle & Johnstone, 2020, p. 6), but this seems somewhat arbitrary, as the brain is probably involved in most, if not all forms of mental disorder (though not necessarily as an active cause).

Based on Brinkmann (2024), I have introduced RDoC and PTMF since they distinguish themselves as two of the most important current critical perspectives on a symptom-focused diagnostic psychiatry. But at the same time, it seems that each of them lacks what the other has to offer. In the rest of this article, I will try to outline a perspective that integrates both the brain and the life contexts in a theory of psychopathology as situated, and I will do so by formulating four tentative principles for such a theory. Neither the brain nor the context are sufficient in themselves, because the situatedness consists precisely in the way in which the two (and other relevant factors) intersect in a person’s life. At least that is the hypothesis to be pursued below.

## A Theory of Psychopathology as Situated is Relational

This is perhaps the most important point because it underscores the importance of describing psychopathology neither as a purely brain/personal matter (cf. RDoC) nor a purely environmental/contextual matter (cf. PTMF). The fact that psychopathology is situated surely does not mean that the person’s contribution to influencing the environment is neglected. That something is situated means instead that all relevant factors that contribute to creating a situation in a person’s life must be considered, which also includes the person’s brain, body, and experiences that matter when they are brought into the world to create a situation. A situation is necessarily a situation for *someone* that lives a life in a world. The medical sociologist Gannik (2005) expressed it in a clear and simple way in her work on a situational theory of illness, where illness is never solely a property of a person or of the environment, but always of the relationship between the two. According to Gannik, this means that we must abandon any essentialism about what illness is. On the one hand, we must see the concept of illness as relational, i.e. “identical with a person’s interactive relationship with her surroundings” (p. 332), and also as something performative: “The theory abstains from approaching illness as something ‘in itself’ beyond those actions or reactions with which a person responds to everyday, bodily experiences” (p. 332). For Gannik, illness is something people “do” in relation to physical and social environments. This does not mean that it can be removed volitionally, but it does mean that disease exists in its manifest appearance when individuals interact and act in environments. Gannik’s field of research was somatic medicine (particularly back disorders), but her theory seems even more relevant to describing psychological problems, where the relational and performative aspects are at least as salient (this does not mean that people are responsible for their disorders, because they could just “do” them differently, but rather that suffering is found in the way it appears in people’s life contexts).

Another emphasis on the relational can be seen in the influential work of Jerome Wakefield. His theory of mental illness is called the “harmful dysfunction” theory and has two components that are related (Wakefield, 1992): In order to talk about mental illness/disorder, there must first be something that is harmful. You are therefore only mentally ill if you experience suffering and discomfort to a sufficient extent. According to Wakefield, this is a value component in the theory, as he believes that social norms and values ​​determine both what counts as “adequate” and what is normally done to help and relieve. These norms vary historically and culturally. But in addition to this value component, there is also a factual component with regard to dysfunction. You are not mentally ill just because you suffer (since suffering can arise from all kinds of problems and life situations), but only if the suffering is connected to a dysfunction in the person’s mental mechanisms. Wakefield here draws on evolutionary psychology, which argues for the existence of genetically based mental modules in the form of innate psychobiological mechanisms in analogy to physiological mechanisms in the body’s organs. Just as a heart is dysfunctional when it becomes unable to pump blood around the body, a mental module is similarly dysfunctional when it causes a person to constantly feel anxiety, for example, without any imminent danger. It is not pathological to feel strong anxiety if you are a paratrooper who is about to be dropped behind enemy lines, but if you experience the same discomfort in harmless everyday situations, and if this discomfort is due to defective mental modules, then, according to Wakefield, we can talk about anxiety as a mental disorder.

Gannik’s theory is situated because it places psychopathology in a relationship between person and environment, and Wakefield’s theory places psychopathology in a relationship between a dysfunctional mental component in the person and an environment that causes the person to suffer, for example due to a lack of help and support. A mental disorder is therefore only present when both sides of the relationship meet in *a situation*: A harmful environment and a dysfunction in the individual. An individual dysfunction can be caused by environmental stressors and adversities, just as adversities may arise because of an individual’s dysfunctions. But a dysfunction in a supportive environment does not lead to psychological disorder (e.g., because it is compensated for), and a harmful environment without a dysfunction does not cause psychological disorder either (but nevertheless experienced hardship, suffering, or discomfort). It remains for proponents of the theory to explain in greater detail what can be meant by a “mental module”, but regardless of whether this can be specified satisfactorily or not, it is intuitively plausible that a situated understanding must involve a relation between a person-component and a context component, which are not two immutable elements, but which instead form part of a transactional process that forms the situation.

## A Theory of Psychopathology as Situated Needs a Concept of Ecosocial Niches

The next point concerns the specification of the situated– what does it mean to say that something is situated? A well-founded proposition, rooted in evolutionary biology, is that psychopathology, like all other kinds of life processes, is situated in an econiche. Recent research has developed a concept of neuroecosocial niches (Rose et al., 2022) that is particularly relevant to psychology and a theory of psychopathology. In the word lies a unification of the neural, the ecological and the social. It is brains, physical environments, and social relationships that together form a niche as the basis of life for people. The concept of niche can be defined in many ways, but the authors write: “We think of niches as both relational– established in relation to other niches– and substantive, in that a niche implies a certain mode of life of the organism within a specific habitat in an ecological system.” (p. 124). In evolutionary biology, the term is typically used to denote the reciprocal relationship between the organism and the environment. On the one hand, a niche is the basis of life for the organism– e.g. a spider’s web or the earthworm’s soil– and on the other, this basis of life is itself influenced by and in some cases created through the organism’s own activity. The spider makes the web itself, in which it climbs around and uses as a net to catch insects, and the soil is a waste product from the earthworm’s absorption of organic material (e.g., dead insects and leaves), which in itself provides fertile ground for the growth of new plants, and this is how the ecological cycle runs to create the niche (Costall, 2004).

In biology, the theory of niche construction emphasizes the fact that organisms are not just passive victims of selection pressure, but themselves take part in shaping environments that favor certain traits. With this perspective– which is sometimes called mutualism (Still & Good, 1998)– we thus get a far more dynamic view of the relationship between organism and environment, which becomes even more tangled when it comes to humans, who as cultural beings in a unique sense live in an econiche that is produced through social practice. In a human way of life, niche construction occurs when norms and knowledge are materialized in everything from buildings, databases, libraries and to the Internet, which are handed down across generations in apprentice-like relationships (Sterelny, 2012). An econiche is therefore normative in that it tells the participants how the world should be approached, something which ecological psychology has conceptualized as affordances (normatively preferred possibilities for action as materialized in relation to the environment). People are introduced to and brought up to be able to perceive, understand and reproduce these affordances in their econiches. Affordances give people reasons for action relative to the social practices that are performed within niches. But these processes can also go wrong or become problematic, and some researchers have begun to analyze mental disorders as inflexible forms of action that go against a person’s own interests within a niche or network of affordances (Nielsen, 2022).

To help alleviate psychopathology, from this perspective, it would be helpful to create niches that are flexible and inclusive for people (Krueger, 2022). The concept of niche has the advantage that it emphasizes how the person herself is an actor in her own life who can influence her own life conditions, while at the same time maintaining that there can be different and unequal access to resources for niche construction. As Rose and Fitzgerald (2022) point out, we must avoid making individuals responsible for their own “biosocial fate” (p. 206), while we should at the same time keep in mind that people are not simply passive victims of the world around them but can actively transform the environment based on their own interests (p. 185). Precisely the concept of niche is meant to capture this duality (that the person both creates and is created in an environment), and there is now a great deal of important work to be done in interpreting people’s mental disorders in the light of problematic relationships situated within ecosocial niches.

## A Theory of Psychopathology as Situated has an Externalist Component

In recent years, many perspectives have emerged within psychology and neighboring sciences that have criticized the classical cognitivist information processing paradigm, i.e., the whole view of the mind as a kind of machine that processes internal, mental representations of the external world according to specific syntactic rules or algorithms (Brinkmann, 2018b). As an alternative to this, an overarching approach known as the 4E paradigm has become influential. The four E’s stand for the mind being embodied, embedded, enacted, and extended. Of course, not all researchers in the field subscribe to all principles. The four E’s can be translated to mean that the mind is corporeal, i.e., not merely localized in the brain, but functionally linked to a living, sentient, moving body (cf. Merleau-Ponty, 1945); that mental functions are necessarily situated in a concrete context where people live, act, and experience (cf. Wittgenstein, 1953); that mental life is linked to action and activity and not primarily come from a passive observer (cf. Dewey, 1896; Gibson, 1986); and finally– as regards the externalist component– that what we call a person’s mind is extended beyond brain and body through all kinds of external mediators including various forms of technology (Rowlands, 2010).

One of the best-known proponents of the externalist point of view today is the philosopher Andy Clark, who claims that we humans are “natural born cyborgs” (Clark, 2004). We use things and technologies to expand and refine our mental processes. Everything from glasses that help us see more clearly to scientific instruments, books, diagrams, etc. are involved in human cognition as an intrinsic part of mental life. Clark’s theory can more accurately be called “active externalism” (Clark & ​​Chalmers, 1998), as it focuses on how persons and surroundings are actively and mutually involved in cognitive and more broadly psychological processes. The conclusion is, in short, that cognitive processes are not in the head– at least not exclusively. A simple example highlighted by Clark is a notebook. If a patient with incipient Alzheimer’s manages to cope with everyday life by jotting down relevant information in a notebook, then the notebook is of course a substitute for the “brain power” that previously helped the person to remember, but there is no reason to believe that the notebook is less psychologically relevant simply because it is an external thing outside of the skull. If a person asks the patient if he knows the height of Mt. Everest, and the patient says yes, but can only answer by consulting the notebook, which correctly says “8.848 meters”, then we have no reason to deny that the person really knew it. In that example, the notebook plays the same functional role for the person as “brain-based” memory plays for the people whose brains function in such a way that they can use it (the brain) to remember the height of mountains. A person who does not suffer from Alzheimer’s may at first have difficulties remembering it and say “Wait… I’ll just think about it… yes, now I have it: 8.848 meters!”. It is correct to say that the person always knew this fact, just as it is correct to say that the patient, who has to look up the notebook to remember it, always knew about the same fact (Brinkmann, 2018b).

For the externalist perspective on the mind, it is a challenge that it becomes difficult to delimit the bounds of mental life, when suddenly everything can be functionally included in a person’s life processes. But if we perceive the mind as abilities and dispositions to recognize features of the world, solve problems, act and respond emotionally to what happens (Brinkmann, 2018b), then from an externalist and broader 4E perspective, one will focus on the mediators that enable these abilities and dispositions. Some of them belong to the brain, others to the body, social life and the physical world consisting of, among other things, things and technologies. I believe that this is a very promising perspective in terms of general psychology, but it is still only sparingly applied to an understanding of psychopathology. However, an article by Sneddon (2002) can be mentioned, which argues for an externalist view of certain mental disorders; and also Hoffman (2016), who examines the implications of the perspective for psychiatry; and Stotz (2014), who connects the externalist 4E perspective to an evolutionary understanding of the framework, where it is cultural and not just genetic inheritance that is at the center. Again, we have a nascent paradigm waiting to be developed in relation to psychopathology as situated.

## A Theory of Psychopathology as Situated Sees the Brain as a Social Organ

I have argued that a theory of psychopathology as situated must be relational, since it is the relationship between personal and environmental factors that is constitutive of mental disorders. This relationship can favorably be conceptualized via the biological niche concept, which for humans is not just about a simple relationship between the organism and the world, since it also involves culture, technology and sociality as mediators of this relationship. Furthermore, such a theory, in line with the 4E perspective, should have an eye for the external factors, since mental disorders not only concern the intra-cranial, but also factors outside the person’s brain and body. I have previously tried to analyze ADHD from this perspective (Brinkmann, 2016b). Finally, I want to return to the brain, which deserves a more thorough treatment, as it is of course involved in all forms of mental life, including mental illness and disorder, but it is important to describe its role correctly, so as to avoid reducing mental life to processes in the brain.

Thomas Fuchs, a phenomenological psychiatrist, has made a valuable contribution to the description of the brain as a social organ relevant to a theory of psychopathology as situated (Fuchs, 2009, 2018, 2021). Fuchs subscribes to an externalist and ecological view of the psyche and the brain (Fuchs, 2009). He argues that mental life is linked to both brain and body and can be described by three cycles: One that is about organismic self-regulation; one that concerns relations between organism and environment; and one that concerns intersubjective interactions. He writes that the brain is a social and biographical organ that is part of all these cycles. Not as the place where the mind is located, because it makes no sense to say where the mind “is”, but as an essential component of a person’s interaction with the world: The mind, according to Fuchs, is not located in any one place at all, but distributed among the brain, the body and the world, and thus continually crosses the borders of the skull. (Fuchs, 2009, p. 229). Psychopathology, in this light, is best understood as breakdowns or inadequacies in the ways in which the cycles function and in which the brain forms a central part as a social organ. Fuchs (2021) therefore calls for a “relational medical science” and a “person-centered psychiatry” that simultaneously focuses on biological processes, psychopathological experiences, biographical connections and social interactions rather than reducing the perspective to what goes on in the brain. The brain is involved as a relational and social organ in all these processes, but always as a person’s organ and not the other way around: Being a sensing, feeling, acting, suffering person is not a product of brain activity, but the brain should instead be thought of as the person’s organ that enables these psychological functions and sometimes obstructs them. You can also simply say that the brain for Fuchs is a situated organ that is physically located in a person’s head and body, but which is also the person’s mediator for all possible life processes. In this way, Fuchs avoids committing what is known as the “mereological fallacy” of attributing abilities and dispositions to the brain that only make sense when attributed to the organism as a whole.

Many recognized researchers commit the mereological fallacy (see the scathing critique in Bennett & Hacker, 2003). Mereology is the study of the relationships between parts and wholes, and the mereological fallacy consists in attributing properties to a part of something (e.g., the brain) that can only meaningfully be attributed to the whole (e.g., the living organism). When someone claims that the brain can be aware, think, feel, remember, sense, etc., they make this mistake. Because in reality the brain cannot be aware of anything or perceive anything. What it can do is transform energy, and its energy transformation obviously has a bearing on how a person can be aware or perceive something, but that does not change the fact that it is the person and not the brain that can do these things. The fact that the brain cannot pay attention does not mean that it is inattentive. That the brain cannot see does not mean that it is blind– just as my sandwich cannot be said to be either awake or asleep! This is not an empirical fact about brains and sandwiches, because we cannot one day discover that the brain can actually see, or that my sandwich can actually sleep. It is instead what Wittgenstein called a grammatical fact, i.e., a conceptual fact about how we can meaningfully apply the psychological terms of language. Wittgenstein expressed the point thus: “only a living human being and what resembles it (behaves in a similar way) can be said to have sensations; that it sees; is blind; belongs; is deaf; is conscious or unconscious.” (Wittgenstein, 1953: § 281). Our mental concepts can only be applied to living beings with a certain behavior - and the brain is not a being in that sense, but an organ, and it does not behave in any way at all, but is the seat of complex neurochemical processes, which help to enable people to perform actions. In this way, the brain is more like a tool used by a person in their situated life activities than it is a seat of mental processes (Harré, 2012; Brinkmann, 2018b). And, as a tool, it is extremely relevant to an understanding of psychopathology, as the tool can be dysfunctional or decidedly defective. It may be here that Wakefield’s somewhat speculative theory about mental modules can find a more secure foundation if the theory is connected to neuroscientific knowledge about the brain. In that case, there may be a legitimate biologization of our understanding of the mind (Brinkmann, Birk & Lund, 2023). It may also be the case that some brains function differently than the average, as claimed by the neurodiversity movement. The implications of this are yet unknown, but certainly worth delving into further for a theory of psychopathology as situated (Leadbitter et al., 2021).

## Conclusion

In the first part of the article, I reviewed the central problem of the traditional psychiatric understanding of mental disorder, which is based on a non-situated perspective that gives rise to the VSAD fallacy– i.e., the view of symptoms as diseases in themselves, as abstracted from both spatial, temporal, and social contexts. I then introduced two critical perspectives that in each way transcend psychiatry’s attachment to symptoms, namely RDoC with a focus on the brain and PTMF with a focus on the environments that affect people. I argued that a situated perspective is required to get out of the impasse psychiatry has ended up in, where symptoms are seen as mental disorder. Instead, a viable understanding of mental disorder must understand how the person’s brain and environment *both* play together in the constitution of mental life– also when it becomes problematic, dysfunctional, and leads to suffering. From Gannik, Wakefield, and Fuchs in particular, a relational perspective on psychopathology was taken as situated. Not only in the social sense (concerning relationships between people), but also in an ecosocial niche with an externalist component, where the brain is included as a social organ. These four concepts (relation, ecosocial niche, externalism, and the social brain) frame tentative principles for a theory of psychopathology as situated, which of course must stand the test both in terms of research, when human suffering and psychological disturbances are to be understood, and in practice as a framework for the development of interventions that do not just focus on discrete, abstracted symptoms, but views people’s lives as situated. From a situated perspective, it may sometimes be relevant to focus on a person’s brain when mental disorders occur, and at other times it may be more relevant to focus on environmental factors in a treatment context. But in all cases, it will be important to understand the relationship between brain-person-environment, as none of the components can be understood without the others, and this is precisely what a situated understanding of psychopathology will insist on.

## Data Availability

No datasets were generated or analysed during the current study.

## Footnotes

1 This article is a based on a paper in Danish that has been significantly revised and adjusted to the present context; see Brinkmann, S. (in press). Situeret psykopatologi. *Nordiske Udkast*.
